# Fused supernumerary kidney: a clinical image

**DOI:** 10.11604/pamj.2023.44.114.38841

**Published:** 2023-03-01

**Authors:** Sushma Myadam, Revat Meshram

**Affiliations:** 1 Department of Paediatrics, Jawaharlal Nehru Medical College, Datta Meghe Institute of Medical Sciences, Sawangi Meghe, Wardha, Maharashtra State, India

**Keywords:** Supernumerary kidney, ultrasonography, computed tomography

## Image in medicine

A partially fused left supernumerary kidney with separate collecting systems and single ureter is diagnosed by using ultrasonography and multi-phased computed tomography (CT). The CT revealed separate renal arteries supplying both kidneys on the left side. Supernumerary kidney is a rare congenital anomaly; less than 100 cases are reported in medical literature over the years. A fused supernumerary kidney is rarer still. Supernumerary kidney is a third kidney and it is a rare congenital anomaly of the urinary tract. The supernumerary kidney needs to be differentiated from the more commonly occurring duplex kidney, which is defined as having two pelvicalyceal systems that are associated with a single ureter or with double ureters. A few anomalies have also been associated with supernumerary kidneys such as ureteral atresia, vaginal atresia, horseshoe kidney, complete duplication of urethra and penis with ectopic ureteral opening into the vagina or introitus, imperforate anus, ventricular septal defects, meningomyelocele, and coarctation of the aorta.

**Figure 1:**
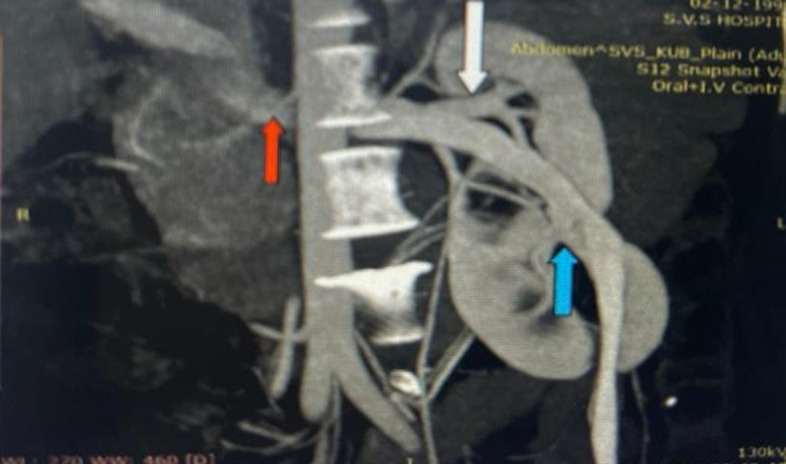
computed tomography showing supernumerary kidney

